# Causal effect of PM_2.5_ on the urban heat island

**DOI:** 10.3389/fdata.2025.1546223

**Published:** 2025-03-14

**Authors:** Yves Rybarczyk, Rasa Zalakeviciute, Marija Ereminaite, Ivana Costa-Stolz

**Affiliations:** ^1^School of Information and Engineering, Dalarna University, Falun, Sweden; ^2^BIOMAS, Universidad de las Américas, Quito, Ecuador

**Keywords:** air quality, urban temperature, causation, statistical inferences, non-linear effects

## Abstract

The planet is experiencing global warming, with an increasing number of heat waves worldwide. Cities are particularly affected by the high temperatures because of the urban heat island (UHI) effect. This phenomenon is mostly explained by the land cover changes, reduced green spaces, and the concentration of infrastructure in urban settings. However, the reasons for the UHI are complex and involve multiple factors still understudied. Air pollution is one of them. This work investigates the link between particulate matter ≤2.5 μm (PM_2.5_) and air temperature by convergent cross-mapping (CCM), a statistical method to infer causation in dynamic non-linear systems. A positive correlation between the concentration of fine particulate matter and urban temperature is observed. The causal relationship between PM_2.5_ and temperature is confirmed in the most urbanized areas of the study site (Quito, Ecuador). The results show that (i) the UHI is present even in the most elevated capital city of the world, and (ii) air quality is an important contributor to the higher temperatures in urban than outlying areas. This study supports the hypothesis of a non-linear threshold effect of pollution concentration on urban temperature.

## 1 Introduction

### 1.1 Global warming and urban impacts

As the World Meteorological Organization (WMO) reports that 2023 has broken the record for the hottest year since preindustrial levels, media have shifted the focus from “global warming” to “global boiling” to emphasize the increasing global temperatures (United Nations News, 2023; World Meteorological Organization., 2024). Additionally, the past 9 years have been the warmest in the 174-year history of recorded observations, with each consecutive decade warmer than the previous one since the 1980s (United Nations, 2024). This marks a significant acceleration in warming trends, coinciding with unprecedented levels of greenhouse gas concentrations, leading to more severe heat waves, storms, and rising sea levels, which are expected to disproportionately impact cities (United Nations Climate Change, 2023). These developments underscore the urgent need for adaptive strategies to mitigate the heightened risks that urban centers face.

As cities grow and the world becomes more urbanized, the Urban Heat Island (UHI) effect is becoming a more pressing issue (Oke, 1973; Zhou et al., 2017). This phenomenon refers to the tendency of cities to experience warmer air temperatures compared to the surrounding countryside (Howard, 1818). While the sun's heat and light reach the earth uniformly, the temperature differences between urban and rural areas are attributed to land cover changes, reduced green spaces, and the concentration of infrastructure in urban settings (Santamouris, 2015). This effect can result in urban-rural temperature differences of 1–2°C during the day and 3–6°C at night (Cichowicz and Bochenek, 2024; Clarke et al., 2022; Santamouris, 2015; Santamouris and Kolokotsa, 2016; Ulpiani, 2021).

The primary health concern associated with the UHI effect is the elevated risk of heat-related illnesses (Heaviside et al., 2017; Thanvisitthpon, 2023). The increased temperatures in urban areas, exacerbated by UHI, can lead to heat stress, heat exhaustion, and heatstroke. Vulnerable populations, such as the elderly, children, and individuals with pre-existing health conditions, are particularly at risk (Anderson et al., 2013; Arsad et al., 2022). Prolonged exposure to extreme heat can strain the cardiovascular system and exacerbate respiratory conditions, posing a significant threat to public health (Anderson et al., 2013; Khatana et al., 2023; Xu et al., 2023).

Air quality in urban settings, often referred to as the Urban Pollution Island (UPI), is frequently compromised by the UHI effect, with higher temperatures contributing to the formation of ground-level ozone and other air pollutants (Ulpiani, 2021). The combination of elevated temperatures and increased air pollution can worsen respiratory conditions such as asthma and chronic obstructive pulmonary disease (Han et al., 2023; Tiotiu et al., 2020; Tran et al., 2023). Poor air quality associated with UHI can also have broader implications for cardiovascular health, potentially increasing the risk of heart attacks and other cardiovascular diseases (Freed, 2023). Additionally, there is a reverse relationship between atmospheric pollution and urban temperature. Previous studies have suggested that aerosol emissions may influence the UHI effect (Piracha and Chaudhary, 2022; Wang et al., 2022a; Ulpiani, 2021; Cao et al., 2016). However, despite the well-documented correlation between UHI and UPI in climatology, their interaction remains comparatively underexplored (Ulpiani, 2021) and no statistical methods have been applied to demonstrate the causal effect of air quality on urban temperature. This work proposes a novel approach grounded in data-driven modeling to address this research gap.

### 1.2 Modeling non-linear dynamics

In general, natural systems are dynamic, multidimensional, and non-linear. As such, typical linear models are not suitable for analyzing the variable interactions in a complex ecological environment (Gregory, 2016). A method that captures the magnitude of correlated dynamic change is needed. For this reason, our approach employs Empirical Dynamic Modeling (EDM), a non-parametric framework for modeling non-linear dynamic systems. Building on the EDM framework, we integrate the Convergent Cross-Mapping (CCM). While EDM offers a foundation for modeling system dynamics, CCM enhances the analysis by providing a quantitative method to define causal relationships among variables (Sugihara et al., 2012). By systematically exploring cross-mapping, we gain insights into the feedback loops and dependencies that shape the system's dynamics. This fusion of EDM and CCM models allows us to explain the nuanced behaviors and causal connections within non-linear systems, leading to a more comprehensive analysis of the complexities inherent in air temperature-aerosols system.

Dynamic causation highlights the intricate relationships between time series variables, which are considered causally related when they are coupled within the same dynamic system; perturbing one variable will consequently affect the other. In this context, if variable X influences variable Y, it follows that Y holds information about X, enabling the prediction or recovery of X from the historical data of Y. This means that the states of X can be effectively reconstructed by analyzing the past behavior of Y, illustrating the interconnectedness of these variables and the potential for utilizing one to infer insights about the other. This relationship can be rigorously tested through cross-mapping, a method that helps validate the causal links between the variables.

The relationship between climate and atmospheric pollution is intricate and mutually dependent. EDM and CCM is a novel non-parametric approach for analyzing this non-linear dynamic system. At present, several studies have explored the EDM-CCM framework, which has found increasing application in various fields, including ecology (Deyle et al., 2016b; Ushio et al., 2018), climate science (Zhaoni et al., 2023), epidemiology (Deyle et al., 2016a; Ma et al., 2017; Rybarczyk et al., 2024), and medicine (Natsukawa and Koyamada, 2017). DeAngelis and Yurek (2015) stated that EDM offers a promising quantitative method for utilizing time series data to create models that can effectively project future dynamics.

Due to its elevated location in the Andean mountains, Quito, the capital city of Ecuador, is used as a unique case study to analyze and understand the link between atmospheric pollution and urban temperature.

## 2 Method

### 2.1 Study site

**Figure 1:**
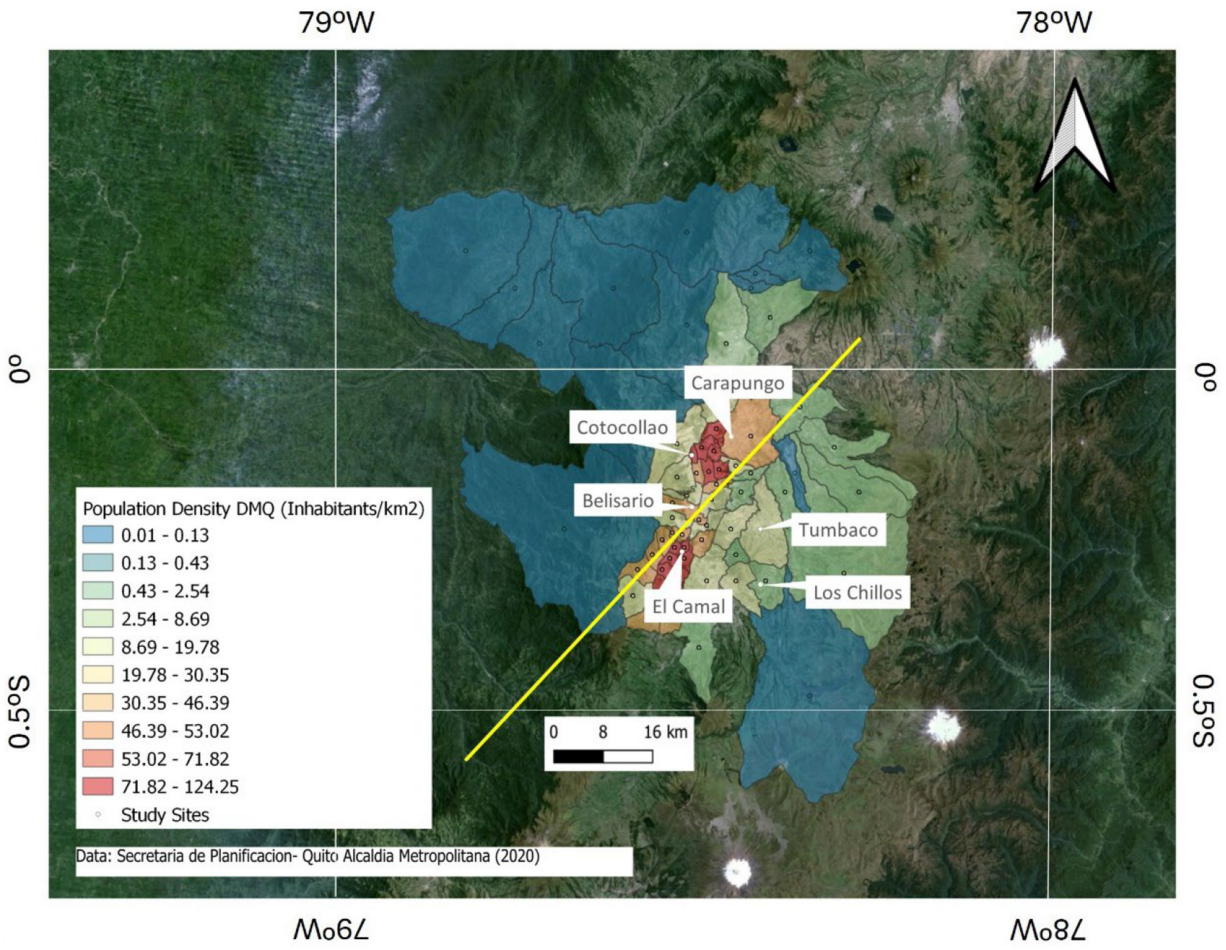
Population density of the Metropolitan District of Quito by parish (2020). The yellow line represents the urbanization axis of the city.

The study focuses on six neighborhoods of the metropolitan district of Quito (DMQ). These districts are divided into two groups: urban and suburban areas. The urban areas are: Belisario (elevation: 2,835 m above mean sea level (m.a.s.l.); coordinates: 78°29′24″W, 0°10′48″S), El Camal (elevation: 2,840 m.a.s.l.; coordinates: 78°30′36″W, 0°15′00″S), and Carapungo (elevation: 2,660 m.a.s.l.; coordinates: 78°26′50″W, 0°5′54″S). And the suburban areas are: Tumbaco (elevation: 2,331 m.a.s.l.; coordinates: 78°24′12.4164″W, 0°12′53.334″S), Los Chillos (elevation: 2,453 m.a.s.l.; coordinates: 78°27′36″W, 0°18′00″S), and Cotocollao (elevation: 2,739 m.a.s.l.; coordinates: 78°29′50″W, 0°6′28″S). These two clusters are objectively defined through the calculation of the urbanization axis (UA) of the city, which exhibits an elongated shape constrained by two parallel mountain ranges, the Western and Eastern Cordilleras (Figure 1). The urban cluster is composed of the three nearest districts to the UA, whereas the suburban cluster is defined by the three furthest districts to this axis. The computation of the UA is carried out by a weighted linear regression of the population density of each parish of the DMQ. One geographic point at the center of each parish is utilized to calculate the regression. Then, the occurrence number (or weight) for each point is determined by the population density of the respective parish. Equation 1 shows the calculation of the weight of each geographic point (*w*_*i*_), which is obtained by dividing the mean parish density (*d*_*i*_) by the mean lowest parish density (*D*).


(1) $$\begin{array}{l} w_{i}=\;\frac{{\bar{d}}_{i}}{\bar{D}} \end{array}$$

The result is an axis stretching from the South-West to the North-East (see yellow line in Figure 1). A perpendicular line to the UA intercepting the location of the monitoring station of each district gives the distance between these two points. The distance between the six monitoring stations and the UA is used to represent the shape of the urban heat island (UHI) for Quito. One suburban (Cotocollao) and two urban (Belisario and Carapungo) stations are located in the North-West of the UA. One urban (El Camal) and two suburban (Tumbaco and Los Chillos) stations are situated in the South-East of the UA.

Quito is characterized by its high-altitude complex terrain. Each neighborhood is situated at varying elevations with a total vertical span of 509 meters between the highest (El Camal-−2,840 m.a.s.l.) and the lowest (Tumbaco−2,331 m.a.s.l.) district. This difference of elevation has an impact on temperature, because the average temperature decreases as altitude increases, making regions at higher elevations colder (Peterson, 2003). The change in temperature with elevation is known as the environmental lapse rate. This variation is in a range of 0.6 to 7.0°C for every 100 meters of elevation gain. Since the average temperature changes can vary based on local conditions, we used 0.7°C/100 m from studies estimating the temperature lapse rate in high-altitude basin (Zhao et al., 2022) and complex terrains (Gao et al., 2012). This correction is applied to the raw measurements, in order to normalize the altitude effect and to allow a fair comparison of temperatures between the six studied areas, for the correlation analysis. No correction of temperature is necessary for the causation analysis, as the EDM-CCM method is based on the time series variation, and not the absolute values, of the studied variables. The characteristic of each monitored site is summarized in Table 1.

**Table 1:** Study areas ranked by descending order of elevation.

**District**	**Type**	**Elevation (m)**	**Mean raw temperature (°C)**	**Mean corrected temperature (°C)**	**Distance from UA (km)**
El Camal	urban	2,840	13.98	14.02	2.37
Belisario	urban	2,835	13.94	13.96	1.34
Cotocollao	suburban	2,739	13.93	13.31	7.06
Carapungo	urban	2,660	14.74	13.61	4.44
Los Chillos	suburban	2,453	15.34	12.86	15.45
Tumbaco	suburban	2,331	16.17	12.89	9.37

**Figure 2:**
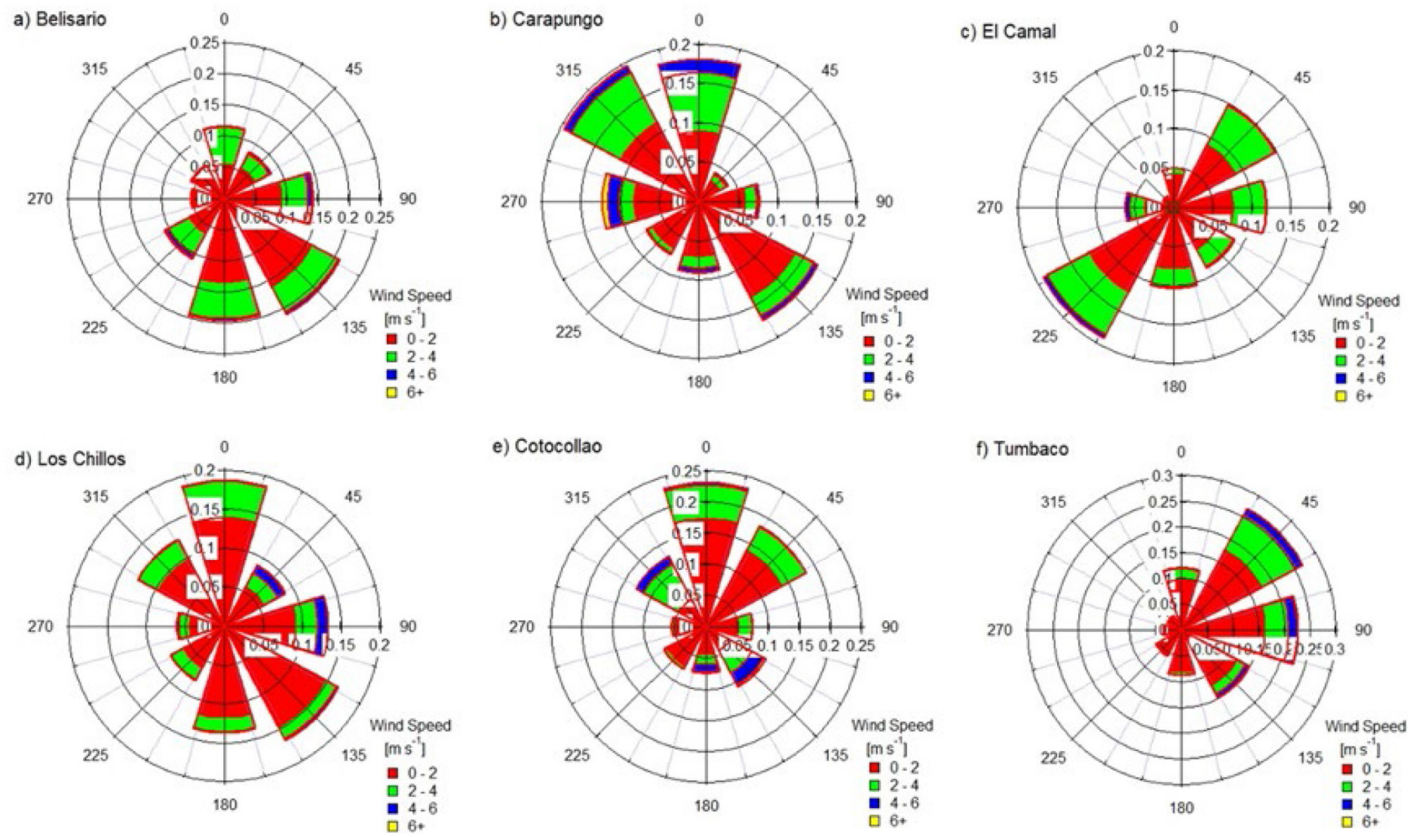
Wind rose plots for six study sites: A) Belisario, B) Carapungo, C) El Camal, D) Los Chillos, E) Cotocollao, and F) Tumbaco.

Figure 2 illustrates the wind rose plots for all study sites. The dominant wind direction may vary from one site to another, which is typical of a montane city where the terrain is complex. Additionally, we note that the wind speed distribution range is relatively low (≤ 6 m/s) across all districts. This suggests that most of the pollution is generated locally rather than being transported from distant sources, and the wind direction would not matter that much, unless it was coming from a nearby road. Furthermore, Ecuador is not an industrialized country, and most urban air pollution originates from traffic activity (Alvarez-Mendoza et al., 2018).

### 2.2 Data collection

The data utilized in this research were collected by the Municipal Office of Environmental Quality in Quito. This involved real-time hourly monitoring over 19 years (from 2004 to 2023), using state-of-the-art Environmental Protection Agency (EPA) standard equipment installed in multiple locations (i.e., six monitoring stations) across the city. Thermo Scientific FH62C14-DHS was used to obtain concentrations of particulate matter with aerodynamic diameter ≤ 2.5 μm (EPA No. EQPM-0609-183). Apart from the air pollution data, meteorological parameters were also measured in the same monitoring stations. For that, completely automatic weather stations were used. Temperature was measured by Thies Clima equipment. The data thus obtained are not only extensive but also characterized by their high fidelity and representativeness of the actual environmental conditions in the neighborhoods of Quito.

To ensure data quality procedures, recommended by the U.S. Environmental Protection Agency (EPA) and the World Meteorological Organization (WMO), internationally calibrated standards from the National Institute of Standards and Technology (NIST) to guarantee data traceability are applied (U.S. Environmental Protection Agency, 2017). First, there is regular staff training to ensure that operational personnel are skilled in handling equipment, performing maintenance, and executing calibration procedures accurately. In addition, Secretariat of Environment performs a preventive maintenance program, to minimize equipment failures and ensures the reliability of monitoring instruments. Furthermore, comprehensive calibration program involves: (i) sampling to ensure that air samples are consistently representative of the monitored environment; (ii) measurements to verify that instruments are accurately capturing air quality and meteorological parameters; and (iii) calibration to regularly adjust instruments against known standards to maintain measurement accuracy. Finally, rigorous acquisition procedure ensures that all monitoring equipment and standards used meet predefined quality criteria. Apart from the equipment checks, daily data monitoring of pollutant trends and urban meteorological parameters allows for the early identification of calibration deviations or potential equipment malfunctions. And continuous automated nightly checks validate that the sampling, measurement, and calibration equipment perform consistently and accurately.

### 2.3 Convergent cross-mapping

The statistical method of convergent cross mapping (CCM) is used to infer a possible causal effect of fine particulate matter on urban temperature. This technique, developed for studying complex dynamical systems, allows us to analyze the relationships between the two time series of PM_2.5_ and temperature. The principle consists of determining whether changes in temperature can predict changes in the concentration of PM_2.5_, thereby providing insights into the directionality and strength of the interaction between the two variables. The method operates on the premise that if the variable PM_2.5_ influences the variable temperature, then the state of temperature can be used to predict the state of PM_2.5_ over time. Conversely, if PM_2.5_ does not influence temperature, knowing temperature would not help in predicting PM_2.5_. CCM is particularly valuable in systems where relationships are non-linear, and traditional correlation or linear regression methods may fail to capture the complexity of the interactions (Sugihara et al., 2012). This is the reason why CCM is most applied in ecology and environmental sciences, particularly in climate studies (Ye et al., 2015; Rybarczyk et al., 2024).

**Figure 3:**
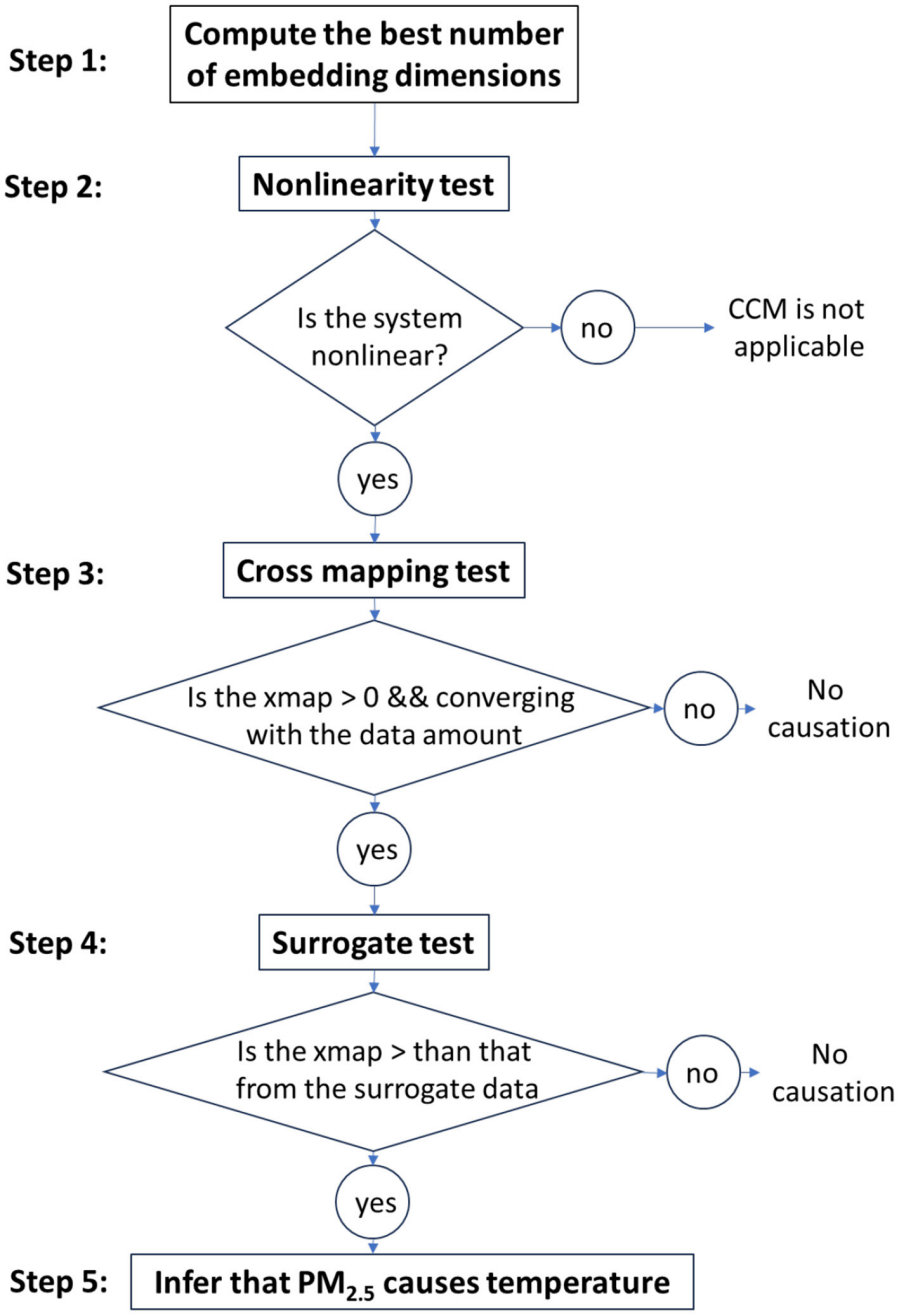
Diagram describing the main steps of the CCM method for inferring a causal relationship between PM_2.5_ and urban temperature.

For applying this powerful tool and making causal inferences, several criteria need to be fulfilled (Figure 3). First, the best number of embedded dimensions must be calculated. The embedding is based on the Takens' theorem to reconstruct the phase space of each time series (Takens, 1981). This involves transforming the time series data into a higher-dimensional space where the dynamics of the system can be described. Second, the non-linearity of the relationship between PM_2.5_ and temperature needs to be tested. An important condition for applying the CCM is to make sure that the analyzed system is deterministic and non-linear (Chang et al., 2017). Third, the cross-map skill (xmap) for predicting PM_2.5_ from temperature must be greater than zero and must increase with the library size (i.e., amount of training data). Finally, a surrogate data test is carried out. The surrogate is generated by a shuffled version of the original time series. If the xmap skill for the original data is significantly better than for the surrogate, it can be concluded that the apparent causality is likely not due to chance. All of these conditions need to be met for inferring a causal effect of the fine particulate matter on the urban heat island (Yuan and Shou, 2022).

The CCM analysis is implemented in R programming language and uses the dedicated library rEDM (Park et al., 2023). The developed script is composed of four main operations. First, the data are loaded as a parameter of the EmbedDimension() function in order to define the best number of dimensions to get the highest predictive skill. Second, the linearity is tested using the PredictNonLinear() function. The non-linearity is established when the highest prediction skill is obtained for a dimensional space higher than zero. Third, the convergent cross-mapping is applied using the best dimension computed in the previous steps and the variables to analyze as parameters. The CCM() method provides the xmap skill as a function of library size. Finally, the significance (*p*-value) of the causation is calculated by comparing the original time series to a surrogate. The surrogate test is conducted to reject the null hypothesis that confounding variables, other than air pollution, may influence the causal relationship with temperature. The “ebisuzaki method” is utilized to create surrogates by randomizing the phases of a Fourier transform, preserving the power spectra of the null surrogates.

## 3 Results and discussion

### 3.1 Correlation analysis

**Figure 4:**
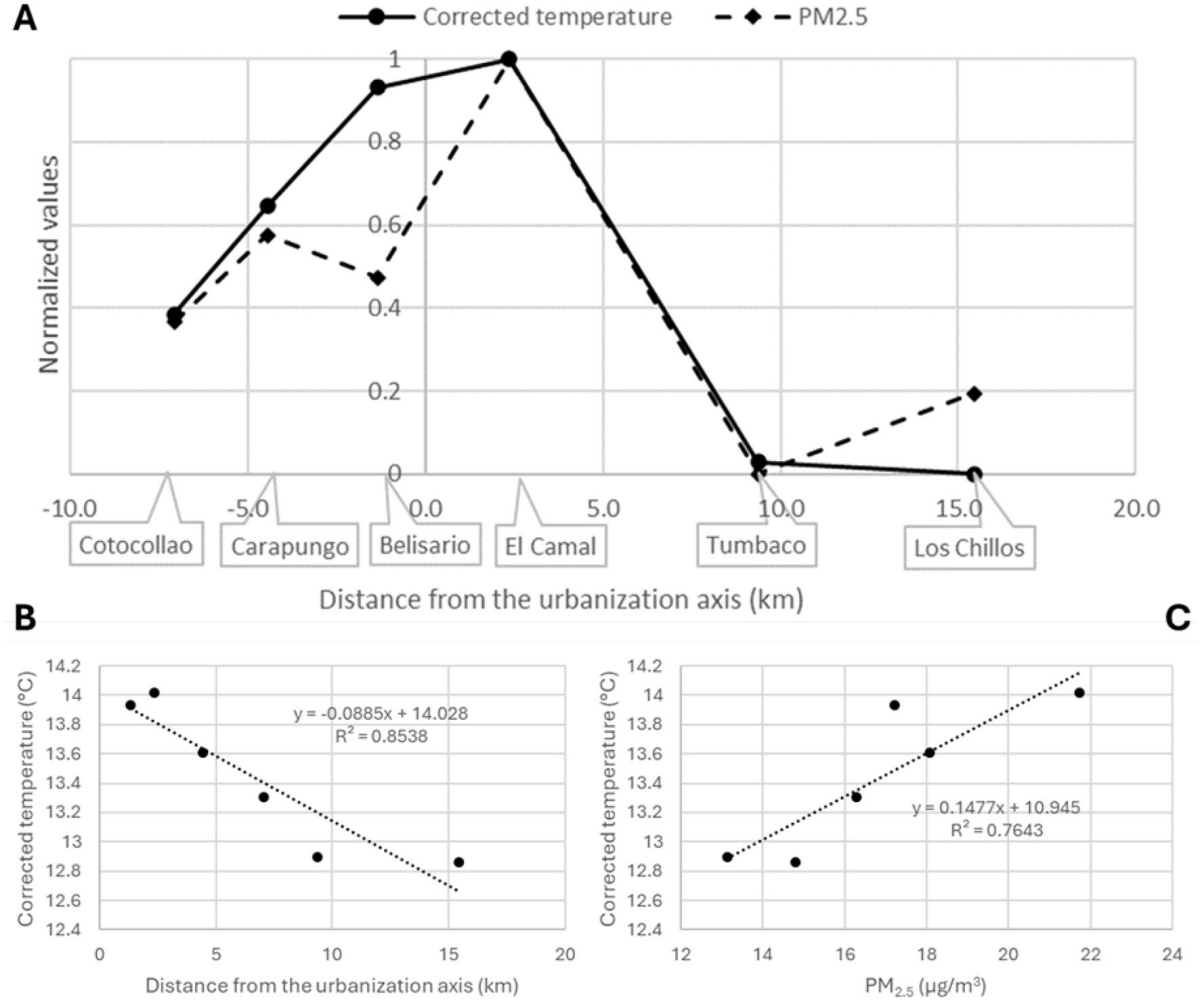
Representation of the UHI and its correlation with air pollution. The normalized values of corrected temperatures (continuous line) and PM_2.5_ concentrations (broken line) are plotted for each monitoring station (panel **A**). Negative kilometers are arbitrary attributed to the North-West districts for displaying the shape of the UHI. Panels **B** and **C** represent the correlation of the corrected temperatures with the distance from the urbanization axis and air pollution, respectively.

The shape of the UHI for the DMQ is represented in Figure 4 (panel A, continuous line). The plot shows clearly that the temperature is affected by the distance from the UA, confirming previous studies indicating warmer city cores (Gartland, 2008; Howard, 1818; Kazakou et al., 2009; Oke, 1973; Tzavali et al., 2015; Zhou et al., 2017). The highest temperatures are observed for the urban districts close to the UA, whereas the lowest temperatures are recorded for the suburban districts. The corrected temperature is particularly low for the two furthest districts to the UA (Tumbaco and Los Chillos). This visual analysis is confirmed by the high correlation (*R*^2^ = 0.854) between the corrected temperatures and the distance from the UA (Figure 4, panel B). A similar profile is observed for the values of the fine particulate matter over the metropolitan district (Figure 4, panel A, broken line). The concentrations in PM_2.5_ are higher for the urban than the suburban areas. As shown before, the urban form, population and density of economic activity directly influence PM_2.5_ concentrations (Wang et al., 2022b). These results are coherent with the expected values of temperature (Orlando and Berazategui, 2024) and air pollution (Zalakeviciute et al., 2018a) in a mid-size South American city.

Figure 4 (panel C) shows that the correlation between the corrected temperature and the concentration of PM_2.5_ is also high (*R*^2^ = 0.764). There is a fair overlapping between the normalized values of temperature and PM_2.5_ (Figure 4, panel A). Only one district (Belisario) has a concentration of fine particulate matter that deviates from the trend of measured temperatures. This difference can be explained by the fact that the monitoring station for this district is located near two big urban parks (i.e., Parque de Mujer and Parque La Carolina), that have been identified as important air pollution filters for the city (Hernandez et al., 2019; Zalakeviciute et al., 2024). This result supports the hypothesis of an impact of the fine particulate matter on the urban temperature. The finding is confirmed by an additional analysis that involved calculating the correlation between temperature and various spatial parameters related to land use and population density (Table 2). The results show that temperature has a stronger correlation with PM_2.5_ (*r* = 0.87) than with urbanization (*r* = 0.66), which rules out the assumption that factors other than air pollution could more effectively explain the urban heat island effect in Quito.

**Table 2:** Correlation (Pearson's coefficient) of temperature with main environmental and demographic variables (refer to Table A1 in the Appendix for data sources).

	**Natural land (%)**	**Urban area (%)**	**Agricultural area (%)**	**Population density (inhab/km^2^)**	**PM_2.5_(μg/m^3^)**
**Temperature** (°C)	−0.65	0.66	−0.60	0.57	0.87

However, the correlation between PM_2.5_ and UHI could be explained by confounding variables that have a causal effect on both air pollution and temperature. Besides urbanization, industrial activity leads to higher emissions of pollutants (Liu et al., 2022) and can also contribute to local warming (Meng et al., 2022) due to human activities. Additionally, socioeconomic growth typically entails increased energy consumption which often relies on the combustion of fossil fuels, resulting in both heat generation (Li et al., 2020) and atmospheric pollution (Feng et al., 2023). Lastly, wind patterns can simultaneously influence PM_2.5_ and temperature. Wind plays a crucial role in dispersing and transporting air pollutants over long distances (Kleine Deters et al., 2017). It can also affect the presence of temperature inversions that trap pollutants close to the ground, especially in urban areas surrounded by mountains, such as Quito (Glojek et al., 2022). Regarding temperature, wind impacts heat distribution, which can either exacerbate or mitigate the UHI effect by influencing local weather conditions (Abbassi et al., 2022). To distinguish causality from spurious correlation, the second analysis consists of performing the CCM on the time series of the PM_2.5_ and temperature variables.

### 3.2 Causal analysis

**Figure 5:**
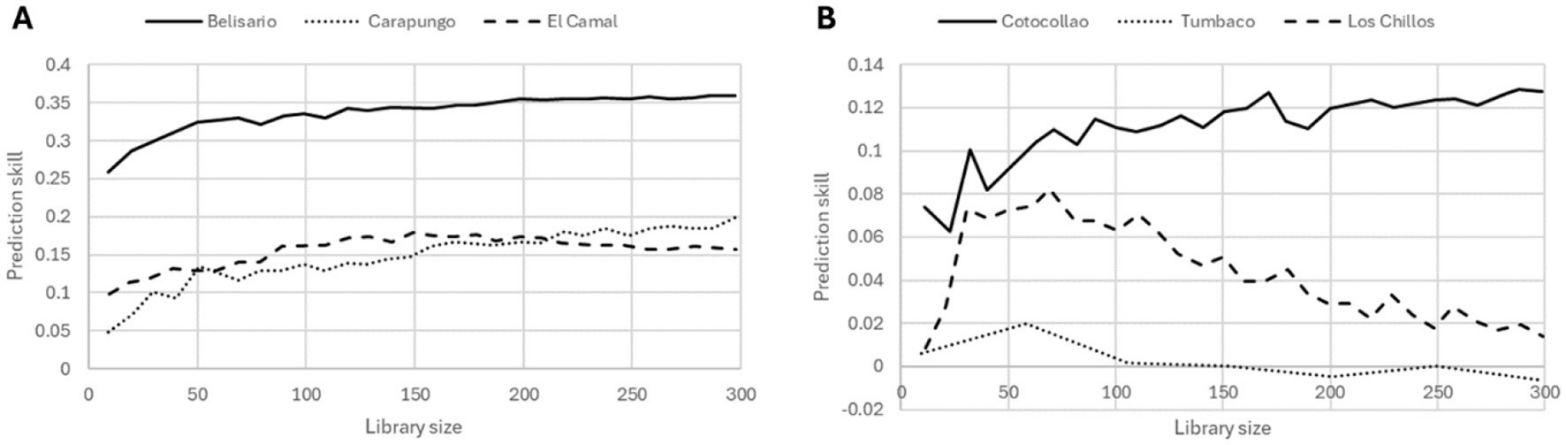
Convergent cross mapping of PM_2.5_ with temperature in urban (panel **A**) and suburban (panel **B**) areas.

The causal analysis shows the best number of four embedded dimensions for the studied sites. The non-linearity test demonstrates that the deterministic dynamic system is non-linear. These two results allow us to apply the CCM method to infer a causal relationship between air pollution and temperature. The CCM exhibits an important difference between the urban and suburban areas (Figure 5). In the case of the three urban districts a similar pattern can be observed (Figure 5, panel B). For all of them, the cross-mapping is positive (prediction skill > 0) and increasing with the library size. This result validates step 3 of the CCM workflow (Figure 3) for the urban sites. On the contrary, the suburban areas present a dissimilar profile of prediction skill (Figure 5, panel B). The xmap for Tumbaco is negative and decreasing with the library size. Los Chilos has a positive xmap, but its value decreases with the amount of training data. Cotocollao is the only suburban neighborhood exhibiting a positive xmap that increases with the library size. The fact that the cross-mapping test (Figure 3, step 3) is not validated for Tumbaco and Los Chillos means that no causal relationship between PM_2.5_ and temperature can be inferred for these two suburban areas. This result is consistent with the fact that these zones are more vegetated, especially the rainy district of Los Chillos, which might have a double effect of mitigating both the particle pollution and heat (Cohen et al., 2014; Xing and Brimblecombe, 2020).

The surrogate test (step 4) shows a significant difference of the xmap between the original dataset and a shuffled version of the time series for one suburban area (Tumbaco) and the three urban areas (Table 3). For the other two suburban areas (Los Chillos and Cotocollao) the *p*-value is above 0.05. This last analysis allows us to conclude on the CCM-based causality of fine particulate matter on temperature. Among the studied sites, only the urban districts meet the three criteria to infer a causation. Consequently, we can deduce that the concentrations of PM_2.5_ have an effect on the measured temperature in the most urbanized areas of the city. For assessing the UHI effect, urban and suburban districts are usually lumped together and compared to the rural areas (Tzavali et al., 2015). Here we show a reduced pollution effect for the suburban neighborhoods in contrast to the urban core. This finding supports the hypothesis of a causal effect of the fine particulate matter on the UHI. The result, along with the correlation analysis, aligns with a previous study pointing out the role of black carbon in increasing the urban temperature (Wang et al., 2022a). This causal relationship can be explained by anthropogenic emissions and radiative forcing effect of black carbon on albedo (Wu et al., 2024; Kopp and Mauzerall, 2010).

**Table 3:** Summary of the tested criteria and causal deductions.

	**Urban areas**			**Suburban areas**		
**Criteria**	**Belisario**	**El Camal**	**Carapungo**	**Tumbaco**	**Los Chillos**	**Cotocollao**
Xmap skill > 0	Yes	Yes	Yes	No	Yes	Yes
Xmap skill ↗ with Lib size	Yes	Yes	Yes	No	No	Yes
Surrogate test (*p*-value)	0.000	0.005	0.023	0.008	0.051	0.499
**Causation**	**YES**	**YES**	**YES**	**NO**	**NO**	**NO**

Since we observe a causal effect of PM_2.5_ on temperature in urban but not in suburban areas, we propose that there may be a threshold effect of anthropogenic emissions contributing to city warming. This observation is consistent with recent studies, which demonstrate varying effects of PM_2.5_ concentration and chemical composition between urban and suburban environments (Wu et al., 2024). Furthermore, these studies highlight the importance of non-linear heating mechanisms, which may depend on urban form (Ming et al., 2023). Our findings, along with these studies, highlight the need for further investigation into the underlying processes driving these patterns, leaving open questions about the specific mechanisms through which PM_2.5_ impacts urban temperatures.

The threshold effect can be understood through the mitigating impact of spatial environment indicators on the interaction between particulate matter and air temperature. Fang and Gu (2022) showed that the significance of the coupling effect between UHI intensity and PM_2.5_ concentrations is characterized by notable spatial heterogeneity. This coupling is influenced by several spatial indicators, including land cover, land use, building forms, and road traffic density. Notably, vegetated areas exhibit a strong negative correlation, suggesting that greenery can mitigate the interplay between UHI and PM_2.5_, thereby reducing urban temperatures. Conversely, urban forms characterized by high building density and various land uses, such as residential and commercial areas, are positively correlated with both temperature and PM_2.5_ levels (Rodríguez et al., 2016; Xu et al., 2017). Additionally, road traffic density contributes further to this effect, releasing heat and pollutants that amplify the interaction between urban warming and air quality degradation (Liu and He, 2012; Husni et al., 2022). Overall, the enhanced human activity in built environments exacerbates the coupling effect between UHI and PM_2.5_, emphasizing the necessity for strategic urban planning that incorporates vegetation and considers land use patterns to alleviate these urban climate challenges.

Some possible limitations need to be discussed before drawing final conclusions from this work. First, it is important to mention that the accuracy of the causal inferences is impacted by the measurement uncertainties. Yuan and Shou (2022) show that CCM performs exceptionally well in a deterministic setting. Conversely, the precision declines when measurement noise is introduced. The greater the noise level, the higher the likelihood of failing to detect causal relationships. However, the probability of this issue is minimal, as data collection is conducted using state-of-the-art equipment that is rigorously calibrated and controlled according to a standard protocol, as outlined in section “2.2. Data Collection.” Furthermore, in the unlikely event that measurement noise impacts our results, it would hide actual causation (false negative) but would not infer incorrect connections (false positive).

A second consideration is the representativeness of the six districts. Although the monitoring stations are geographically dispersed, they cover most of the metropolitan area of Quito. The ability to measure environmental variables from East to West across the city enables us to create a fair UHI profile that encompasses both urban and suburban areas. Measurements in purely rural areas could have enhanced the analysis by providing background concentrations, but no such installations are available in the region. Also, various types of districts are represented in this sampling (Zalakeviciute et al., 2018b). This includes industrial areas, primarily located in the North (Carapungo and Cotocollao) and South (El Camal and Los Chillos) parts of the city, as well as residential areas (Belisario and Tumbaco). Our results indicate that the characteristics of the district (industrial vs. residential) are less correlated with air temperature than with the concentration of PM_2.5_, suggesting that fine particulate matter is a primary driver of the UHI.

Lastly, potential confounding variables pose the main challenge in inferring true causation in uncontrolled settings. Here, meteorological conditions can be identified as a possible confounding variable. However, the results of the causal analysis can rule out this hypothesis. CCM determines whether a variable truly influences another by examining how well the history of one variable (i.e., temperature) can predict the state of another (i.e., pollution). The fact that temperature consistently predicts PM_2.5_ despite changes in meteorological conditions supports the notion that fine particulate matter has a causal effect on air temperature. This cross-mapping between the studied variables helps to distinguish genuine causal relationships from those merely correlated due to confounding influences.

## 4 Conclusions

The Urban Heat Island (UHI) is a complex interplay between urban planning, local climate, and human activities. Due to the fact that the effect of air pollution on this phenomenon is underexplored, we investigated the link between fine particulate matter (PM_2.5_–particulate matter with aerodynamic diameter ≤ 2.5 μm) and urban temperature. The first finding shows that even in the highest capital city of the world (Quito, Ecuador) a clear UHI effect is present. There is a negative correlation between the distance from the urbanization axis and air temperature. The second result reveals a high positive correlation between the concentration of PM_2.5_ and temperature. A fair overlapping between the geographic profile of the air contaminant and temperature is observed. In order to avoid a spurious correlation, a causal analysis based on CCM was performed. The findings underscore the significance of the PM_2.5_ concentrations as a contributor to the city's thermal dynamics. A causal impact of PM_2.5_ on the temperature is confirmed in the urban districts, only. It is probably due to an effective range of pollution concentration, which needs to exceed a certain threshold to affect significantly the city temperature. This outcome aligns with the expected characteristics of the UHI, where urban areas experience higher temperatures compared to their suburban and rural neighborhoods.

While urban districts have more asphalt surfaces, suburban areas feature more dirt or concrete tile roads, which may influence the urban albedo of Quito. Specifically, asphalt and soot from traffic-related PM contribute to a warming effect in urban regions, whereas lighter, more naturally derived PM in suburban areas has a cooling effect (Zereini and Wiseman, 2011). In previous research examining the chemical composition of PM_10_, which also includes PM_2.5_, we observed distinct chemical differences between urban and suburban sites. Specifically, the urban site exhibited more elements related to anthropogenic sources, including black carbon, while suburban PM_10_ was composed of more natural elements (Zalakeviciute et al., 2020). In other words, the distinction between urban and suburban areas can be attributed to the fact that PM_2.5_ primarily originates from dust in suburban regions, while the source of fine particulate matter in urban areas is largely anthropogenic. This human-generated pollution contains black carbon, which warms cities by absorbing sunlight and heats the urban atmosphere (Schmidt, 2011).

Our study supports the idea that minimizing the emission of air pollutants can reduce the UHI effect (Cao et al., 2016; Wang et al., 2021). Addressing the UHI impact is pivotal in the broader context of global warming mitigation. Urban planning strategies, including the expansion of green spaces, promotion of 'cool roofs', or alternative solutions, in addition to enhancement of public transportation, could be instrumental in reducing air temperature of the cities. The present work is the first statistical demonstration of the causal impact of anthropogenic atmospheric emissions on the UHI. Our findings can support the public decision-making process by adopting policies that aim to (i) impose stricter standards for greenhouse effect pollutant emissions and fuel by replacing gasoline-powered cars with hybrid, electric and hydrogen vehicles; and (ii) reflect on urban solutions that mitigate air pollution and elevated temperature to help prevent chronic diseases. Further research is needed to evaluate the long-term effects of green infrastructure and sustainable transportation policies. In-depth studies should also explore the interconnectedness of UHI, atmospheric pollution, climate change, and their collective impact on human health.

## Data Availability

Publicly available datasets were analyzed in this study. This data can be found at: http://aireambiente.quito.gob.ec/.

## Appendix

**Table A1:** Spatial and environmental parameters for each district.

	**Tumbaco**	**Los Chillos**	**El Camal**	**Cotocollao**	**Belisario**	**Carapungo**
**Natural land cover** (%)	6.35	16.16	5.27	5.45	2.45	9.81
**Urban area** (%)	92.91	72.97	94.73	94.73	97.55	90.19
**Agricultural area** (%)	0.73	10.87	0	0	0	0
**Population density** (people/km^2^)	11.2	2.2	85.01	71.97	22.1	30.38
**Mean PM**_2.5_ (μg/m^3^)	13.13	14.80	21.74	21.74	17.21	18.08
